# The biomarkers suPAR and blood eosinophils are associated with hospital readmissions and mortality in asthma – a retrospective cohort study

**DOI:** 10.1186/s12931-019-1234-4

**Published:** 2019-11-15

**Authors:** K. E. J. Håkansson, Line J. H. Rasmussen, Nina S. Godtfredsen, Oliver D. Tupper, Jesper Eugen-Olsen, Thomas Kallemose, Ove Andersen, Charlotte Suppli Ulrik

**Affiliations:** 1 Department of Respiratory Medicine, Copenhagen University Hospital Amager and Hvidovre, Kettegård Allé 30, 2650 Hvidovre, Denmark; 2 Clinical Research Centre, Copenhagen University Hospital Amager and Hvidovre, Hvidovre, Denmark; 3 Department of Psychology and Neuroscience, Duke University, Durham, NC USA; 4 Institute of Clinical Medicine, University of Copenhagen, Copenhagen, Denmark; 5 Emergency Department, Copenhagen University Hospital Amager and Hvidovre, Hvidovre, Denmark

**Keywords:** Prognosis, Biomarker, Eosinophils, Emergency department, Acute admission

## Abstract

**Introduction:**

Prognostic biomarkers in asthma are needed. The biomarker soluble urokinase plasminogen activator receptor (suPAR) has been associated with asthma control and with prognosis in acutely admitted medical patients. We investigated if suPAR and blood eosinophil counts at the time of admission for asthma are associated with readmission and mortality.

**Methods:**

Our cohort comprised 1341 patients (median age 45.3, IQR 30.1–63.1) acutely admitted with a diagnosis of asthma to Hvidovre Hospital, Denmark (November 2013 to March 2017). Patients had suPAR and blood eosinophils measured at admission. Outcomes were 365-day readmission and all-cause mortality. Logistic regression analysis adjusted for age, sex, C-reactive protein, and Charlson comorbidity score was used to assess the association of the two biomarkers with readmission and all-cause mortality.

**Results:**

Compared to event-free patients, patients who were either readmitted (*n* = 452, 42.3%) or died (*n* = 57, 5.3%) had significantly higher suPAR concentrations (*p* < 0.0001) and lower eosinophil counts (*p* = 0.0031) at admission. The highest odds of readmission or mortality were observed for patients in either the 4th suPAR quartile (*p* < 0.0001) or with eosinophil counts < 150 cells/μL at admission. Increasing levels of suPAR were associated with 365-day readmission (OR 1.3 [1.0–1.6]; *p* = 0.05) and mortality (OR 2.9 [1.7–5.1]; *p* = 0.0002). Eosinophil count > 300 cells/μL was significantly associated with lower odds of readmission (OR 0.64 [0.5–0.9]; *p* = 0.005) and lower mortality (OR 0.7 [0.6–0.9]; *p* = 0.0007).

**Conclusions:**

In patients acutely admitted with asthma, elevated suPAR concentrations together with blood eosinophil count < 150 cells/μL at the time of hospital admission were associated with both 365-day all-cause readmission and mortality.

## Introduction

Poorly controlled asthma is associated with impaired quality of life, accelerated decline in lung function, and, not least in more severe cases, repeated hospital admissions and increased mortality. The Global Initiative for Asthma estimates that half of the global asthma population has uncontrolled or only partially controlled disease [1], a risk factor for potentially life-threatening exacerbations. This major challenge with poorly controlled asthma has led clinicians to search for biomarkers to objectively assess disease control and to help identify patients with a high risk of adverse events. While several risk factors for asthma-related death have been identified, such as high short-acting bronchodilator use [1], there has been limited research in biomarkers of uncontrolled disease and risk of repeated hospitalization.

Soluble urokinase plasminogen activator receptor (suPAR) is the soluble form of the urokinase plasminogen activator receptor (uPAR), a three-domain membrane receptor that is expressed on a plethora of cells ranging from mono- and lymphocytes to endothelial and smooth muscle cells. uPAR plays a central role in the plasmin/plasminogen pathway and is important for chemotaxis, immune system activation, and tissue remodeling [2]. suPAR is readily measurable in plasma, serum, and sputum and is considered a nonspecific inflammatory marker in both acute and chronic illness, with associations ranging from Chronic Obstructive Pulmonary Disease to all-cause mortality [3–5].

In asthma, sputum, biopsy, and serum suPAR has previously been shown to be elevated in stable asthma patients in comparison to controls [6–8]. Plasma suPAR has been shown to discriminate between patients with both objective disease control markers (Peak Expiratory Flow (PEF) above or below 80%) and subjective symptom scores (Asthma Control Test (ACT)-score above or below 20) with acceptable sensitivity and specificity in a relatively small cross-sectional study on asthma control [9]. While these two studies set the scene for suPAR research in asthma, there is very limited knowledge concerning suPAR and its relationship to asthma hospitalization and mortality, especially in larger cohorts.

Another, more intensely studied, asthma biomarker is blood eosinophil count. Eosinophilic asthma, often referred to as T_2_ asthma, is the most prevalent asthma phenotype comprising up to 50% of asthma patients [10]. T_2_ asthma is seen over a wide disease spectrum from mild disease to difficult-to-control or severe asthma with high symptom burden and frequent exacerbations [11]. Eosinophilic inflammation in the airways is most accurately assessed by using either airway mucosal biopsies or sputum eosinophils [12]. However, not least for practical reasons, in clinical settings blood eosinophil count is commonly used as a surrogate marker. Previous long-term studies have shown that asthma patients with elevated blood eosinophil count have increased all-cause mortality [13]. On the other end of the inflammatory spectrum in asthma, non-T_2_ asthma is characterized by both neutrophilic and paucigranulocytic forms, driven by neutrophils and/or mixed leukocyte populations [1]. Interestingly, studies suggest that the inflammation reflected in circulating suPAR concentrations in part stems from neutrophil activity [14, 15], commonly considered to be non-T_2_ inflammation. We thus hypothesize that the two biomarkers could be used in conjunction to improve risk stratification in acutely admitted patients with asthma.

Based on a large, unselected cohort of acutely admitted medical patients, we identified those admitted with a diagnosis of asthma, and we tested the hypothesis that suPAR and blood eosinophil count are independent biomarkers associated with both one-year all-cause readmission and all-cause mortality.

## Materials and methods

### Study cohort and data

This study is a registry-based cohort comprising 29,088 medical patients acutely admitted between November 18th 2013 and March 17th 2017, including 1341 with a prevalent diagnosis of asthma. The study was carried out in the Emergency Department (ED), Copenhagen University Hospital Amager and Hvidovre, Hvidovre, Denmark [5]. The ED receives unselected, adult internal medicine patients of all specialties as previously described [5]. suPAR concentrations and blood eosinophil count were routinely measured as part of the standard admission blood tests. Patients were included in this study, if they i) were admitted to the ED during the study period and had a current diagnosis of asthma (i.e., ICD-10: J45-J46 registered as a primary or secondary diagnosis at the index admission or within the past two years), and ii) had suPAR analyzed.

The index admission was defined as the first admission where a patient had suPAR measured within the study period. Biochemistry data was obtained from the electronic hospital database LABKA via the Department of Clinical Biochemistry. Information on hospital admissions, length of stay, readmissions, and diagnoses were obtained from the Danish National Patient Registry. Vital status of patients was obtained from the Danish Civil Registration System [16]. Patients were followed in terms of readmission and death using national registries until June 17th, 2017, ensuring follow-up of a minimum of three months for all patients.

### Measurement of biomarkers

Blood samples were routinely analyzed at the Department of Clinical Biochemistry. Plasma suPAR (D1D2D3 & D2D3 molecules) levels were measured using the suPARnostic AUTO Flex ELISA (ViroGates A/S, Birkerød, Denmark) on an automated Siemens BEP2000 platform according to the manufacturer’s instructions, as described in detail previously [3, 17]. Plasma suPAR concentrations were analyzed within 48 h. Blood eosinophil count was measured using a Sysmex XE5000 analyzer (Sysmex Corporation, Japan; until February 2015) and a Sysmex XN9000 (from February 2015). Blood eosinophil count was analyzed within 3 h. C-Reactive Protein (CRP) was measured using a COBAS 6000 analyzer (Roche Diagnostics, Mannheim, Germany).

### Statistical analysis

The outcomes of interest in this study were acute all-cause readmission and all-cause mortality within 30-, 90-, and 365 days.

Population-specific quartiles for suPAR were calculated. The suPAR quartile cut-offs were: Q1: < 1.9 ng/mL (*n* = 282), Q2: 1.9–2.6 ng/mL (*n* = 266), Q3: 2.61–3.5 ng/mL (*n* = 237), Q4: > 3.5 ng/mL (*n* = 283).

For blood eosinophil counts, cut-offs were arbitrarily defined as low eosinophils (< 150 cells/μL, *n* = 452), medium eosinophils (150–300 cells/μL, *n* = 237), and high eosinophils (> 300 cells/μL, *n* = 334) [13, 18, 19].

Continuous variables are presented as median (interquartile range; IQR) and categorical variables as number (percentage).

Unadjusted and multivariable adjusted logistic regression analyses were used to estimate the association between suPAR or blood eosinophil count and readmission or mortality. suPAR and blood eosinophil count were used as continuous (log2-transformed) or categorized variables (suPAR quartiles and low/medium/high blood eosinophil count groups). Results are presented as odds ratios (ORs) with 95% confidence intervals (CIs). The multivariable logistic regression analyses were adjusted for sex, age, Charlson comorbidity score, and CRP. Additionally, suPAR analyses were adjusted for blood eosinophil count and vice versa. The Charlson comorbidity score was calculated using a SAS macro as previously described [20], with weights updated by Quan et al. [21].

In the analyses of readmission, death before readmission was considered a competing endpoint. Therefore, all patients who died before the end of follow-up were excluded from this analysis regardless of readmission or not.

Cumulative probability for survival and readmission stratified by suPAR quartiles and blood eosinophil count are presented to illustrate the outcome distribution of patients over time. The discriminative ability of suPAR and blood eosinophil count to predict readmission and mortality was analyzed using Area under the Curve (AUC) for Receiver Operator Characteristics (ROC) curves.

SAS Enterprise Guide 7.11 (SAS Institute) and R 3.2.3 (The R Foundation for Statistical Computing) were used for statistical analysis. R 3.2.3 was used to create the Figs. A *p*-value < 0.05 was considered to be statistically significant.

### Ethics

The study was approved by the Danish Health and Medicines Authority (ref. 3–3013-1061/2) and the Danish Data Protection Agency (ref. HVH-2014-018, 02767).

### Data sharing

The datasets used and/or analyzed during the current study are available from the authors upon reasonable request, but analysis may require approvals from the Danish Patient Safety Authority and the Danish Data Protection Agency.

## Results

**Fig. 1:**
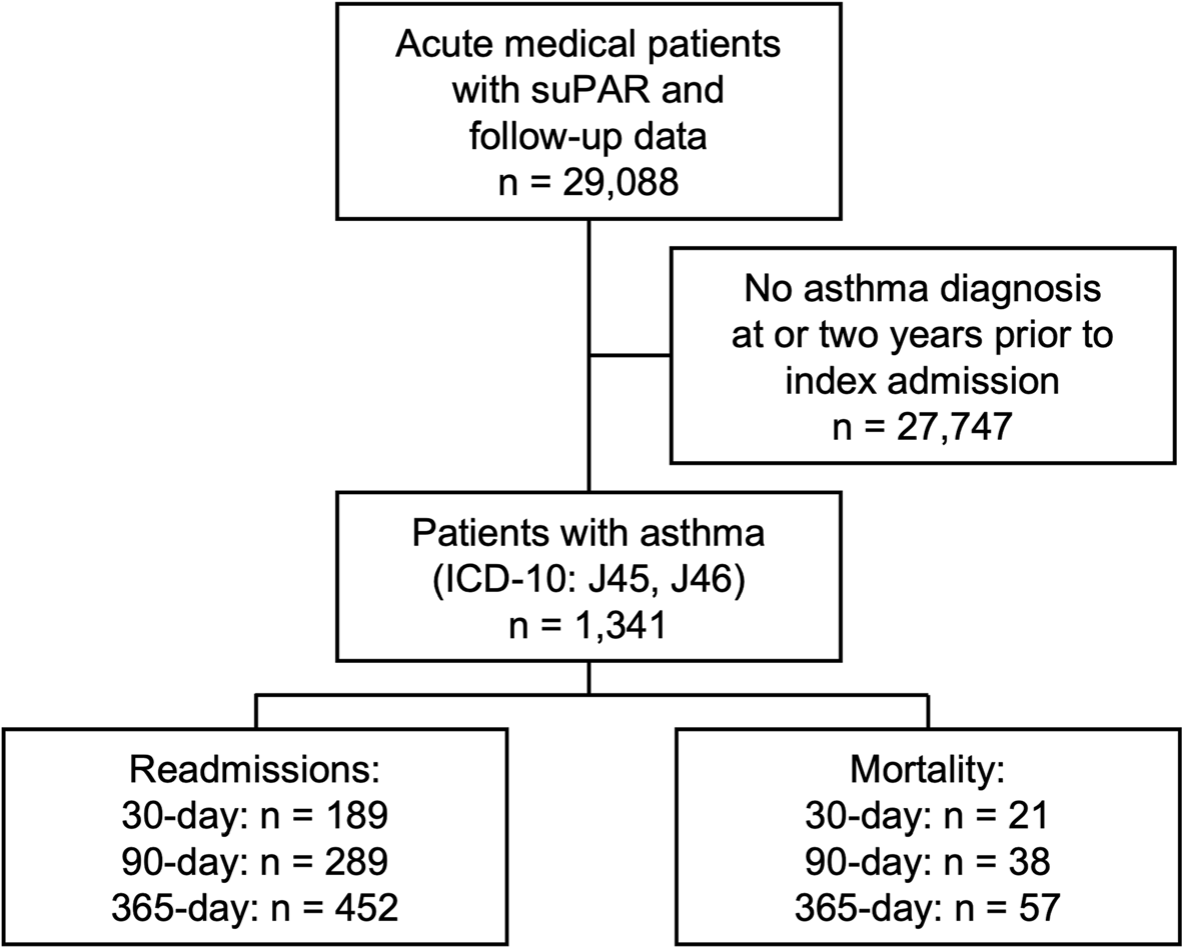
Flowchart of study design and participant endpoints.

Of 29,088 medical patients acutely admitted to the ED at Hvidovre Hospital during the study period, 1341 patients had a prevalent asthma diagnosis and were included in present study cohort (Fig. 1). The median age was 45.3 years, 66% of the admitted patients were female, and the median Charlson score was 1.0 (1.0–1.0) (Table 1).

**Table 1:** Baseline characteristics of 1341 patients with acute admission to hospital and a diagnosis of asthma

	N (%) or median (IQR)
*N*	1341
Men	457 (34.1%)
Age (years)	45.3 (30.1–63.1)
Charlson comorbidity score	1.0 (1.0–1.0)
Length of hospital stay (days)	0.55 (0.27–2.21)
C-Reactive Protein (mg/L) (*n* = 1272)	6.0 (2.0–23.0)
Blood eosinophil count (cells/μL) (*n* = 1268)	180 (60–400)
suPAR (ng/L)	2.6 (1.9–3.5)

IQR – Interquartile Range

suPAR – Soluble Urokinase Plasminogen Activator Receptor

### Readmission and mortality

Out of the 1341 patients admitted with asthma, all-cause readmission was observed for 189 (14.1%) patients within 30 days, 289 (21.6%) within 90 days, and 452 (42.3%) within 365 days. The corresponding numbers for all-cause mortality were 21 (1.6%) within 30 days, 38 (2.8%) within 90 days, and 57 (5.3%) within 365 days, respectively (Table 2).

**Table 2:** Event rates and odds ratios^*^ for readmission and mortality within 365 days in patients with asthma (*n* = 1341) following an acute hospitalization, stratified by suPAR quartiles

Follow-up	Variable	Readmission			Mortality		
Analysis	suPAR^†^	Events (%)^|^	OR (95% CI)	*P*-value	Events (%)^|^	OR (95% CI)	*P*-value
30 days	Continuous		1.36 (1.0–1.8)	0.042		1.85 (0.8–4.4)	0.171
Adjusted	1st quartile	35 (12.4%)	1		1 (0.4%)	1	
	2nd quartile	36 (13.5%)	1.00 (0.7–1.7)	0.99	2 (0.8%)	N/A^§^	
	3rd quartile	40 (16.9%)	1.14 (0.7–1.9)	0.62	1 (0.4%)	0.34 (0.02–6.4)	0.47
	4th quartile	78 (27.6%)	1.96 (1.1–3.3)	0.014	17 (6.0%)	0.95 (0.08–11.2)	0.97
	Total^¶^	189 (41.8%)			21 (36.8%)		
90 days	Continuous		1.26 (1.0–1.6)	0.08		2.79 (1.5–5.3)	0.0019
Adjusted	1st quartile	63 (22.3%)	1		1 (0.4%)	1	
	2nd quartile	61 (22.9%)	1.00 (0.7–1.5)	0.99	2 (0.8%)	N/A^§^	
	3rd quartile	61 (25.7%)	1.06 (0.7–1.6)	0.81	6 (2.5%)	1.63 (0.2–15.8)	0.67
	4th quartile	104 (36.7%)	1.58 (1.0–2.5)	0.049	29 (10.2%)	2.39 (0.3–21.3)	0.44
	Total^¶^	289 (63.9%)			38 (66.7%)		
365 days^‡^	Continuous		1.27 (1.0–1.6)	0.052		2.94 (1.7–5.1)	0.0002
Adjusted	1st quartile	105 (37.2%)	1		2 (0.7%)	1	
	2nd quartile	107 (40.2%)	1.03 (0.7–1.5)	0.88	4 (1.5%)	0.19 (0.02–2.3)	0.19
	3rd quartile	104 (43.9%)	1.26 (0.8–1.9)	0.25	9 (3.8%)	1.58 (0.3–8.6)	0.60
	4th quartile	136 (48.1%)	1.56 (1.0–2.4)	0.044	42 (14.8%)	2.75 (0.5–13.9)	0.22
	Total^¶^	452 (100%)			57 (100%)		

^*^Multivariable adjusted analyses included age, sex, Charlson comorbidity score, blood eosinophil count and CRP

^†^suPAR quartile cut-offs: Q1: < 1.9 ng/mL (n = 282), Q2: 1.9–2.6 ng/mL (n = 266), Q3: 2.61–3.5 ng/mL (n = 237), Q4: > 3.5 ng/mL (n = 283)

^‡^Only patients with a minimum of 365 days follow-up included in analyses (*n* = 1068)

^§^Calculation not possible due to too few events

^|^Shown as number of patients with event (percentage out of total patient population in each group of blood eosinophil count (low, medium, high) at 365 days)

^¶^Total number of events at follow-up as a percentage of number of events at 365 days

CRP – C-Reactive Protein

CI – Confidence Interval

OR – Odds Ratio

suPAR - Soluble Urokinase Plasminogen Activator Receptor

### suPAR in acutely admitted asthma patients

The median suPAR level was significantly elevated at the time of the index admission in patients who were readmitted within 365 days compared to patients without readmissions. Higher readmission rates were observed especially in the 4th suPAR quartile (Table 2). A weak interaction (coefficient estimate 0.15, standard error 0.07, *p* = 0.037) between suPAR and blood eosinophil counts was found at 30 days, but not at 90- nor 365 days (data not shown).

Continuous suPAR concentrations were associated with increased odds of readmission in uni- and multivariable models at 365 days. Odds of all-cause readmission reached 1.56 (95% CI 1.0–2.4; *p* = 0.044) after 365 days in the 4th quartile when compared to the 1st suPAR quartile using the multivariable model adjusted for sex, age, Charlson comorbidity score, CRP, and blood eosinophil count. (Table 2).

**Fig. 2:**
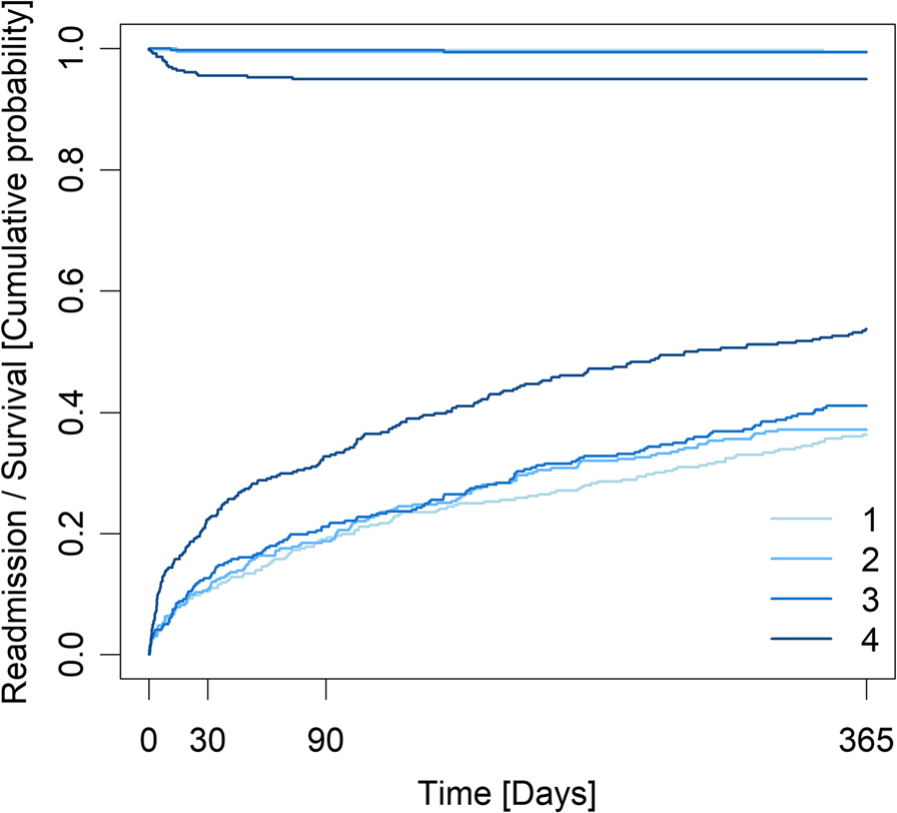
Cumulative incidence plot of mortality (top) and readmission (bottom) within 365 days, stratified by quartiles of suPAR, for 1341 patients acutely admitted with asthma.

Median admission suPAR concentrations were significantly elevated in patients who died, compared to survivors for all three time-points (365 days: 5.1 ng/mL (IQR 3.4–7.1) vs 2.4 ng/mL (IQR 1.8–3.1); *p* < 0.0001) (Table 3). When stratified into quartiles, the highest mortality rates were observed in the 4th suPAR quartile (Fig. 2).

**Table 3:** suPAR concentrations and blood eosinophil count in 1341 patients with asthma following an acute hospital admission

	No event	Readmitted	*P*-value	Died	*P*-value
30-day follow-up					
suPAR (ng/mL)	2.5 (1.9–3.3)	3.1 (2.1–4.1)	< 0.0001	5.6 (3.6–7.7)	< 0.0001
Blood eosinophil count (cells/μL)	190 (70–420)	160 (60–340)	0.088	20 (0–50)	< 0.0001
90-day follow-up					
suPAR (ng/mL)	2.5 (1.9–3.3)	2.8 (2.0–3.9)	0.0002	5.7 (3.5–7.7)	< 0.0001
Blood eosinophil count (cells/μL)	200 (70–430)	150 (60–320)	0.024	30 (10–150)	< 0.0001
365-day follow-up^*^					
suPAR (ng/mL)	2.4 (1.8–3.1)	2.6 (2.0–3.7)	< 0.0002	5.1 (3.4–7.4)	< 0.0001
Blood eosinophil count (cells/μL)	200 (80–450)	160 (60–340)	0.0031	30 (10–130)	< 0.0001

Values presented as percentages or median (interquartile range)

^*^Only patients with 365 days follow-up included in analyses (*n* = 1068)

suPAR - Soluble Urokinase Plasminogen Activator Receptor

Continuous suPAR was associated with increased mortality at 365 days after adjusting for sex, age, Charlson comorbidity score, CRP, and blood eosinophil count: OR 2.94 (95% CI 1.7–5.1; *p* = 0.0002). Although 73.7% of all asthma patients who died belonged to the 4th suPAR quartile at 365 days follow-up (Table 2), there was no statistically significant association between suPAR quartiles and mortality after adjusting for sex, age, Charlson comorbidity score, CRP, and blood eosinophil count (Table 2).

ROC curve analyses showed an AUC of 0.53 (95% CI 0.50–0.56) for suPAR in readmission prediction and 0.84 (95% CI 0.79–0.89) in mortality prediction at 365 days, see Additional file 1: Supplemental AUC graphs for readmission and mortality.

### Blood eosinophil count in acutely admitted asthma patients

**Fig. 3:**
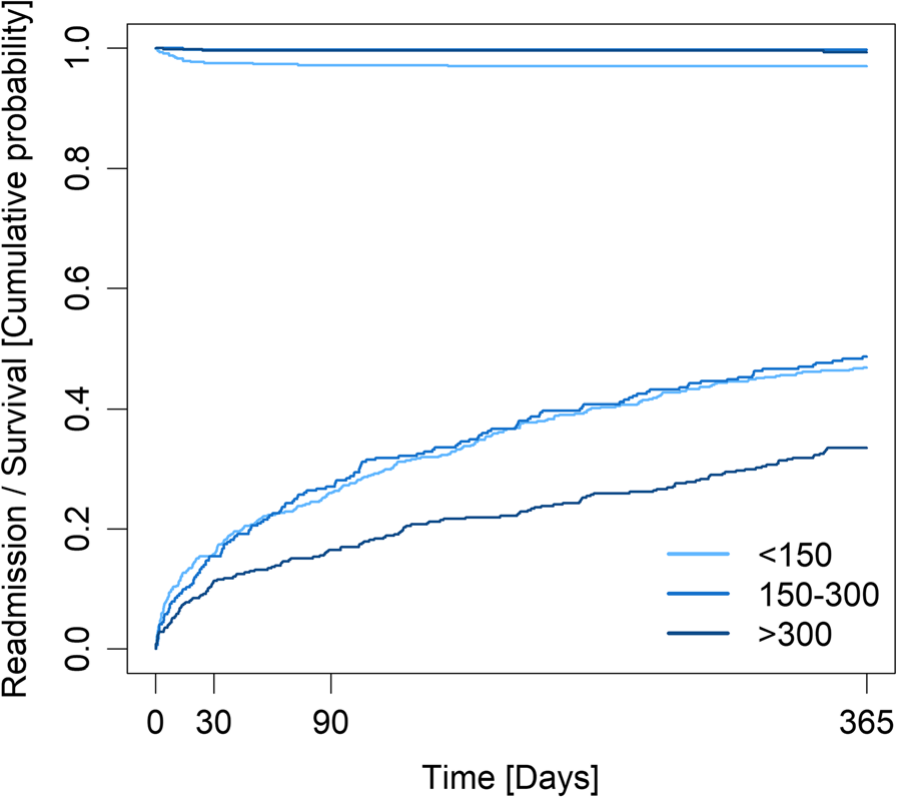
Cumulative incidence plot of mortality (top) and readmission (bottom) within 365 days, stratified by blood eosinophil counts, for 1023 patients acutely admitted with asthma.

Patients with low blood eosinophil count at admission had significantly higher readmission rates at 365 days (Table 3, Fig. 3).

The lowest odds of 365-day all-cause readmission were found in patients with blood eosinophil count above 300 cells/μL (OR 0.64 [95% CI 0.50–0.90]; *p* = 0.005), when compared to patients with blood eosinophil counts below 150 cells/μL in multivariable, adjusted regression analyses (Table 4).

**Table 4:** Event rates and odds ratios^*^ for readmission and mortality within 365 days in patients with asthma following an acute hospitalization, stratified by blood eosinophil count (n = 1268)

Follow-up	Variable	Readmission			Mortality		
Analysis	Eosinophils	Events (%)^‡^	OR (95% CI)	*P*-value	Events (%)^‡^	OR (95% CI)	*P*-value
30 days	Continuous		0.98 (0.9–1.1)	0.64		0.77 (0.5–1.1)	0.137
Adjusted	Low	87 (19.2%)	1		14 (3.1%)	1	
	Medium	45 (19.0%)	0.99 (0.7–1.5)	0.94	1 (0.4%)	0.26 (0.03–2.1)	0.21
	High	47 (14.1%)	0.75 (0.5–1.1)	0.16	3 (0.9%)	0.73 (0.2–2.9)	0.65
	Total events^§^	179 (40.6%)			18 (33.3%)		
90 days	Continuous		0.95 (0.9–1.0)	0.21		0.79 (0.6–1.0)	0.035
Adjusted	Low	129 (28.5%)	1		26 (5.8%)	1	
	Medium	75 (31.6%)	1.15 (0.8–1.6)	0.43	5 (2.1%)	0.48 (0.2–1.5)	0.21
	High	68 (20.4%)	0.66 (0.5–0.9)	0.019	4 (1.2%)	0.41 (0.1–1.3)	0.12
	Total events^§^	272 (61.7%)			35 (64.8%)		
365 days^†^	Continuous		0.94 (0.9–1.0)	0.11		0.70 (0.6–0.9)	0.001
Adjusted	Low	207 (45.8%)	1		41 (9.1%)	1	
	Medium	113 (47.7%)	0.99 (0.7–1.4)	0.94	8 (3.4%)	0.42 (0.2–1.0)	0.061
	High	121 (36.2%)	0.64 (0.5–0.9)	0.0051	5 (1.5%)	0.25 (0.09–0.7)	0.008
	Total events^§^	441 (100%)			54 (100%)		

^*^Multivariable adjusted analyses included age, sex, Charlson comorbidity score, suPAR and CRP

^‡^Shown as number of patients with event (percentage out of total patient population in each group of blood eosinophil count (low, medium, high) at 365 days)

^†^Only patients with a minimum of 365 days follow-up included in analyses (n = 1068)

Cut-offs defined as Low (< 150 cells/μL, n = 452), Medium (150–300 cells/μL, n = 237), and High (> 300 cells/μL, n = 334)

^§^Total number of events at follow-up as a percentage of number of events at 365 days

CRP – C-Reactive Protein

CI – Confidence Interval

OR – Odds Ratio

suPAR – Soluble Urokinase Plasminogen Activator Receptor

Concerning 365-day mortality, the median blood eosinophil count was significantly lower in patients who died compared to survivors (30 cells/μL [IQR 10–130] vs 200 cells/μL [IQR 80–450]) (Table 3). When stratified according to blood eosinophil count, a higher mortality rate was seen in patients with low blood eosinophil count at all time points. Patients with low blood eosinophil count comprised 75.9% of deaths at 365 days (Table 4).

In continuous multivariable regression analyses, higher blood eosinophil count was associated with lower odds of death at 365 days (OR 0.70 [95% CI 0.6–0.9]; *p* = 0.0007). The lowest odds of death at 365 days was seen in patients belonging to the patient group with high blood eosinophil count with OR 0.25 (95% CI 0.09–0.70, *p* = 0.008) (Table 4).

ROC curve analyses showed an AUC of 0.53 (95% CI 0.50–0.56) for blood eosinophil count in readmission prediction and 0.72 (95% CI 0.65–0.79) in mortality prediction after 365 days, see Figs. E1 and E2 in the online data supplement.

### Combining suPAR and blood eosinophil measurements

When event rates are stratified across both suPAR quartiles and groups of low/medium/high blood eosinophil count, an even distribution of patients was seen in terms of readmission (Table 5). However, a 23.4-fold (95% CI 3.2–165.8-fold) increase in relative risk seen for patients in the low blood eosinophil count group between patients in the 1st and 4th suPAR quartiles. In contrast, the mortality in the 4th suPAR quartile was only slightly elevated if patients had a high blood eosinophil count (Table 5).

**Table 5:** Total number of events within 365 days follow-up in a cohort of patients acutely hospitalized with asthma (*n* = 1023), stratified by suPAR quartiles and blood eosinophil count

	suPAR			
Blood Eosinophils	1st Quartile	2nd Quartile	3rd Quartile	4th Quartile
Low (N)^*^	109	104	92	147
No event, N (%)	61 (56.0)	42 (40.4)	51 (55.4)	50 (34.0)
Readmitted^†^, N (%)	47 (43.1)	59 (56.7)	35 (38.0)	66 (44.9)
Mortality, N (%)	1 (0.9)	3 (2.9)	6 (6.5)	31 (21.1)
Intermediate (N)^*^	67	52	60	58
No event, N (%)	40 (59.7)	30 (57.7)	25 (41.7)	21 (36.2)
Readmitted^†^, N (%)	27 (40.3)	21 (40.4)	33 (55.0)	32 (55.2)
Mortality, N (%)	0 (0)	1 (1.9)	2 (3.3)	5 (8.6)
High (N)^*^	101	98	68	67
No event, N (%)	70 (69.3)	72 (73.5)	36 (52.9)	30 (44.8)
Readmitted^†^, N (%)	30 (29.7)	26 (26.5)	31 (45.6)	34 (50.7)
Mortality, N (%)	1 (0.9)	0 (0)	1 (1.5)	3 (4.5)

^*^This analysis only included patients with full 365 days follow-up and blood eosinophil results available (n = 1023)

^†^Only including readmission events in patients without consequent death in the duration of the follow-up period

suPAR quartile cut-offs: Q1: < 1.9 ng/mL (n = 282), Q2: 1.9–2.6 ng/mL (n = 266), Q3: 2.61–3.5 ng/mL (n = 237), Q4: > 3.5 ng/mL (n = 283)

Blood eosinophil count cut-offs defined as Low (< 150 cells/μL, n = 452), Medium (150–300 cells/μL, *n* = 237), and High (> 300 cells/μL, n = 334)

suPAR – Soluble urokinase plasminogen activator receptor

## Discussion

In patients with an active diagnosis of asthma acutely admitted to a hospital, this study shows that the inflammatory biomarkers suPAR and blood eosinophil count are associated with hospital all-cause readmission and all-cause mortality. The highest odds of both readmission and mortality were seen in patients with suPAR concentrations > 3.5 ng/mL and in patients with low blood eosinophil counts (< 150 cells/μL).

### suPAR in acutely admitted asthma patients

While the plasmin/plasminogen-pathway has been extensively investigated in basic research studies, little is known about the mechanistic association between asthma and suPAR. Both in vitro and in vivo studies of acute and chronic disease suggest that neutrophils are an important source of suPAR, as uPAR is shed from the surface of neutrophils, T-cells, and a subset of monocytes upon activation [14, 15, 22, 23]. In asthma, neutrophils and T_1_/T_17_ T-cells are thought to be part of the pathogenesis of the non-T_2_ asthma endotypes [24] which are overrepresented in patients with severe asthma [10, 25], and a previous study has suggested that the uPAR pathway associates to non-T_2_ asthma [7].

Previous studies have shown that suPAR is associated with disease progression and severity, i.e., suPAR levels increase with higher disease stages and increasing severity. We have previously shown in unselected acute medical patients that suPAR levels increase with number and severity of comorbidities (Charlson score) [3] and with deviating vital signs (national early warning score) [26]. However, so far only one published study has explored suPAR’s role in asthma outcomes [9]. Ivanscó and colleagues [9] found that suPAR concentrations were increased in outpatient asthma patients with poor disease control and impaired lung function when compared to patients with well-controlled asthma. In 38 asthmatic patients, a suPAR level of > 4.04 ng/mL identified uncontrolled disease defined as either PEF < 80% of predicted or an ACT score > 20. Furthermore, Ivanscó et al. found no differences in suPAR concentrations between healthy controls and asthma patients with well-controlled disease [9].

We hypothesized suPAR concentrations to be elevated in hospitalized asthma patients due to the systemic inflammation associated with asthma, especially in uncontrolled disease [1]. In the present study, we found generally low suPAR concentrations in event-free patients, which is in line with previous studies in healthy controls [27]. Median suPAR concentrations in event-free patients (2.4 ng/mL) were well below the cut-off for uncontrolled disease suggested by Ivanscó et al., while the majority of readmissions and deaths in our study was observed in patients in the 4th suPAR quartile (> 3.5 ng/mL). As such, the findings of Ivanscó and colleagues regarding disease control in outpatient asthma patients [9] are to some extent transferable to our cohort of acutely hospitalized asthma patients, as they are highly likely to have uncontrolled disease [1].

Mortality rates at 365 days were slightly higher than previous studies on all-cause mortality in asthma patients in Denmark [28], perhaps due to the usage of unselected patients and no age limitations, as previous studies on European populations show a higher mortality rate in older patients [29].

### Blood eosinophil count in acutely admitted asthma patients

The usefulness of blood eosinophil count as a biomarker for acute eosinophilic inflammation is still debated, and while eosinophils are extensively investigated in mechanistic studies, blood eosinophil count is commonly used as a surrogate marker for eosinophilic airway inflammation. The usage of blood eosinophil count as a surrogate marker is not without controversy, as some studies have found weak correlations between blood eosinophils and the gold standard sputum eosinophils [30], while others have found blood eosinophil counts to be accurate in predicting sputum eosinophils in the extreme ends of blood eosinophil count (< 90 and > 400 cells/μL) [31, 32].

The relationship between blood eosinophil counts and hospitalization rate has been thoroughly investigated in recent years, with some studies [18, 33] showing elevated blood eosinophil counts associated to a higher exacerbation rate, while others report conflicting observations [34, 35]. Indeed, diverging results are also seen in terms of blood eosinophil counts and their association to hospital readmission [19, 36].

Regarding mortality, our study showed that the median blood eosinophil count of patients who died was 30 cells/μL and 75.9% of all patients who died had a blood eosinophil count of < 150 cells/μL. The present study thus adds to the growing body of evidence that non-T_2_ asthma is relatively treatment refractory, resulting in frequent readmissions and death, perhaps due to a less efficient response to the initial in-hospital treatment in comparison with patients with normal or elevated blood eosinophil counts [37–40].

Our study showed that in patients with low blood eosinophil counts, 31.9% of readmissions and 75.6% of mortalities belonged to the 4th suPAR quartile, suggesting that combined measurement of the two biomarkers upon admission potentially could improve risk assessment, especially in terms of all-cause mortality. With regards to inflammatory profiles, suPAR might reflect current non-T_2_ inflammation activity, while blood eosinophil counts represent T_2_ inflammation, but further studies regarding the different inflammatory mechanisms and their association to adverse outcomes in asthma are needed.

### Limitations

A limitation to the present study is the lack of information on patient smoking status, as suPAR is known to be affected by smoking [41]. Smoking is also associated with poor disease control and therapy-resistant asthma [42]. Furthermore, in this study based on data from national registries, information on disease severity such as Forced Expiratory Volume (1 s), PEF and ACT-scores, as well as adherence was unavailable. Another limitation is the lack of a validation cohort of acutely admitted patients with asthma to support our findings, however, several studies on the prognostic role of suPAR in general and patient populations support our findings [3, 43]. Due to few deaths, the confidence intervals for mortality estimates were large, meaning greater uncertainty about the exact value of the estimate. Additionally, when stratified according to suPAR and blood eosinophil count, the very low counts are likely not representative of the actual counts in a larger population. The patient inclusion for this study relies on the registration of the diagnosis of asthma in the National Patient Registry. The Danish patient registries used are validated and diagnoses are submitted upon hospital discharge, but some misdiagnoses are inevitable and some patients with asthma might have been missed. Furthermore, some degree of asthma/chronic obstructive lung disease misclassification cannot be excluded. All analyses were based on a single measurement of suPAR and blood eosinophil count, and any changes due to treatment or disease progression could not be assessed.

## Conclusion

In conclusion, elevated suPAR concentration and low blood eosinophil count associated with poor 365-day prognosis in this cohort of acutely admitted asthma patients. Usage of the two biomarkers at the time of acute hospitalization could allow for more precise identification of patients with a high risk of adverse events and potentially also patients with low disease control. However, further research is needed, especially effect studies of treatment on suPAR concentrations in asthma patients.

## Supplementary information

**Additional file 1: Fig. S1*.*** AUC-graph for suPAR and blood eosinophil count-based 365-day Readmission Prediction **Fig. S2*.*** AUC-graph for suPAR and blood eosinophil count-based 365-day Mortality Prediction.

## Data Availability

The datasets used and/or analyzed during the current study are available from the authors upon reasonable request, but analysis may require approvals from the Danish Patient Safety Authority and the Danish Data Protection Agency.

## Abbreviations

ACT: Asthma Control Test
AUC: Area Under the Curve
CI: Confidence Interval
CRP: C-Reactive Protein
ED: Emergency Department
IQR: Interquartile Range
OR: Odds Ratio
PEF: Peak Expiratory Flow
ROC: Receiver Operating Characteristics
suPAR: Soluble Urokinase Plasminogen Activator Receptor
uPAR: Urokinase Plasminogen Activator Receptor
