# Supplementary material for: The biomarkers suPAR and blood eosinophils are associated with hospital readmissions and mortality in asthma – a retrospective cohort study
Source: Respir Res. 2019 Nov 15;20:258. doi: 10.1186/s12931-019-1234-4 (PMC6858677; doi:10.1186/s12931-019-1234-4)
Supplement: Supplementary file 1 — Additional file 1: Fig. S1. AUC-graph for suPAR and blood eosinophil count-based 365-day Readmission Prediction Fig. S2. AUC-graph for suPAR and blood eosinophil count-based 365-day Mortality Prediction. [file 12931_2019_1234_MOESM1_ESM.docx]

**ONLINE DATA SUPPLEMENT**

The Biomarkers suPAR and Blood Eosinophils are Associated with Hospital Readmissions and Mortality in Asthma – A Retrospective Cohort Study

Kjell E. J. Håkansson^1^, Line J. H. Rasmussen^2,3^, Nina S. Godtfredsen^1,4^, Oliver D. Tupper^1^, Jesper Eugen-Olsen^2^, Thomas Kallemose^2^, Ove Andersen^2,4,5^, Charlotte Suppli Ulrik^1,4^

**Figure E1***- AUC-graph for suPAR and blood eosinophil count-based 365-day Readmission Prediction*


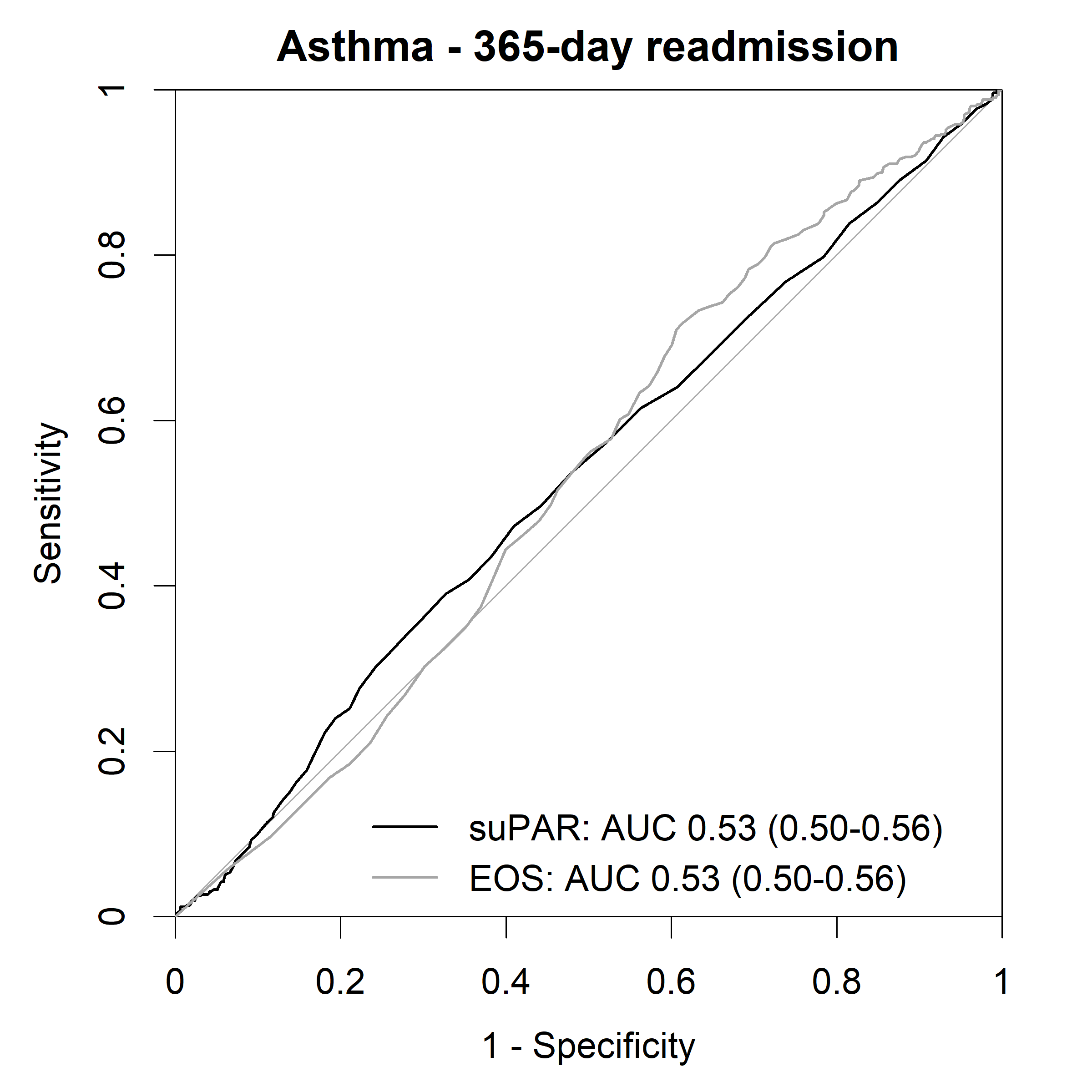


Receiver Operating Characteristic-analysis of suPAR and blood eosinophil count for 365-day readmission prediction in 1,341 patients acutely hospitalized with asthma.

**Figure E2***- AUC-graph for suPAR and blood eosinophil count-based 365-day Mortality Prediction*


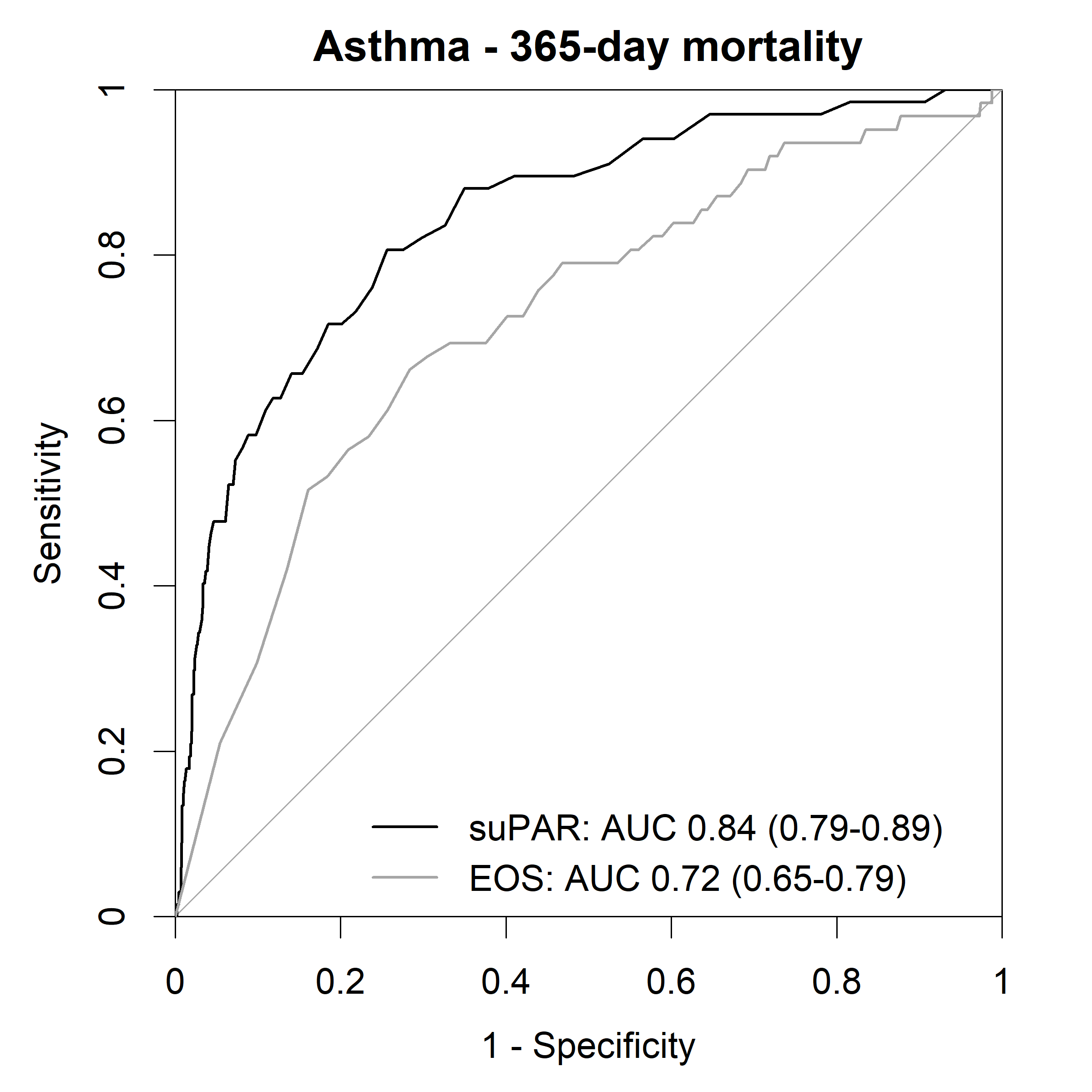


Receiver Operating Characteristic-analysis of suPAR and blood eosinophil count for 365-day mortality prediction in 1,341 patients acutely hospitalized with asthma
